# GSNFS: Gene subnetwork biomarker identification of lung cancer expression data

**DOI:** 10.1186/s12920-016-0231-4

**Published:** 2016-12-05

**Authors:** Narumol Doungpan, Worrawat Engchuan, Jonathan H. Chan, Asawin Meechai

**Affiliations:** 1 Biological Engineering Program, Faculty of Engineering, King Mongkut’s University of Technology Thonburi, Bangkok, Thailand; 2 The Centre for Applied Genomics, Genetics and Genome Biology, The Hospital for Sick Children, Toronto, ON Canada; 3 Data Science and Engineering Laboratory, School of Information Technology, King Mongkut’s University of Technology Thonburi, Bangkok, Thailand; 4 Department of Chemical Engineering, Faculty of Engineering, King Mongkut’s University of Technology Thonburi, Bangkok, Thailand

## Abstract

**Background:**

Gene expression has been used to identify disease gene biomarkers, but there are ongoing challenges. Single gene or gene-set biomarkers are inadequate to provide sufficient understanding of complex disease mechanisms and the relationship among those genes. Network-based methods have thus been considered for inferring the interaction within a group of genes to further study the disease mechanism. Recently, the Gene-Network-based Feature Set (GNFS), which is capable of handling case-control and multiclass expression for gene biomarker identification, has been proposed, partly taking into account of network topology. However, its performance relies on a greedy search for building subnetworks and thus requires further improvement. In this work, we establish a new approach named Gene Sub-Network-based Feature Selection (GSNFS) by implementing the GNFS framework with two proposed searching and scoring algorithms, namely gene-set-based (GS) search and parent-node-based (PN) search, to identify subnetworks. An additional dataset is used to validate the results.

**Methods:**

The two proposed searching algorithms of the GSNFS method for subnetwork expansion are concerned with the degree of connectivity and the scoring scheme for building subnetworks and their topology. For each iteration of expansion, the neighbour genes of a current subnetwork, whose expression data improved the overall subnetwork score, is recruited. While the GS search calculated the subnetwork score using an activity score of a current subnetwork and the gene expression values of its neighbours, the PN search uses the expression value of the corresponding parent of each neighbour gene. Four lung cancer expression datasets were used for subnetwork identification. In addition, using pathway data and protein-protein interaction as network data in order to consider the interaction among significant genes were discussed. Classification was performed to compare the performance of the identified gene subnetworks with three subnetwork identification algorithms.

**Results:**

The two searching algorithms resulted in better classification and gene/gene-set agreement compared to the original greedy search of the GNFS method. The identified lung cancer subnetwork using the proposed searching algorithm resulted in an improvement of the cross-dataset validation and an increase in the consistency of findings between two independent datasets. The homogeneity measurement of the datasets was conducted to assess dataset compatibility in cross-dataset validation. The lung cancer dataset with higher homogeneity showed a better result when using the GS search while the dataset with low homogeneity showed a better result when using the PN search. The 10-fold cross-dataset validation on the independent lung cancer datasets showed higher classification performance of the proposed algorithms when compared with the greedy search in the original GNFS method.

**Conclusions:**

The proposed searching algorithms provide a higher number of genes in the subnetwork expansion step than the greedy algorithm. As a result, the performance of the subnetworks identified from the GSNFS method was improved in terms of classification performance and gene/gene-set level agreement depending on the homogeneity of the datasets used in the analysis. Some common genes obtained from the four datasets using different searching algorithms are genes known to play a role in lung cancer. The improvement of classification performance and the gene/gene-set level agreement, and the biological relevance indicated the effectiveness of the GSNFS method for gene subnetwork identification using expression data.

## Background

The identification of the gene biomarker from high throughput gene expression data for complex diseases is a challenging task. By using high throughput gene expression data, single genes, gene sets, or gene subnetworks have been used as biomarkers from different prior works [1]. Statistical analysis was proposed to identify the disease gene biomarker in consideration of the expression data in different conditions. Since different genetic mutations and dysfunctions of different biological processes are present in complex diseases like cancer, the gene biomarker identification is a non-trivial task. The hallmark of cancer includes sustaining proliferative signalling, evading growth suppressors, resisting cell death, enabling replicative immortality, inducing angiogenesis, and activating invasion and metastasis [2]. Genetic alteration in cancer is one of the factors that gives rise to the cancer complexity. The altered genes are functionally linked in a common biological pathway, enabling tumour cells to activate a specific set of cellular processes also known as the hallmarks of cancer [2, 3]. The alteration on these processes result in the changing of cellular homeostasis and cancer development.

Genes expressed statistically significant among different conditions, such as case and control expression profile, resulted in single gene biomarkers and were inefficient when applied to distinguished disease sample from a healthy sample. The artefacts of microarray data, based on experimental and data processing techniques, resulted in inefficiency of those single gene biomarkers. In order to address this problem, an approach combining gene function related data was introduced.

Subramanian et al. [4] introduced the gene-set enrichment analysis to deal with a large amount of genes in microarray data. The gene sets obtained were groups of genes sharing features in common biological function, chromosomal location, or regulation within each group. Methods of integrating gene-set data with expression profile to achieve the disease gene biomarker as a potential gene representative for a specific function were proposed. Lee et al. [5] and Sootanan et al. [6] proposed methods for gene identification based on gene-set data to obtain genes for pathway activity transformation; this had a high performance on disease classification. However, the identified gene-set biomarkers were unable to illustrate the relationship among these genes for disease mechanism interpretation. Then in order to infer the interaction among those significant genes and imply an insight on the disease mechanism, the network data was integrated with gene expression data to identify the gene network biomarker, which also increases the robustness of the identified gene biomarkers when applying the gene biomarker to another dataset of the same disease.

Several network-based methods were proposed for the gene network termed the subnetwork biomarker. The network data (e.g. gene-gene interaction or GGI, protein-protein interaction or PPI) provided the interaction aspect of gene and protein at systems level. The GGI data provided the relationship of genes in functional related aspects, e.g. the two genes interacted with each other to represent the activation or inhibition of a particular biological process [7]. The expression data were used to infer their interaction among gene pairs by correlation analysis. From the correlation of each gene pair, a gene co-expression network, whose interaction pairs having high correlation may potentially play a role in the same biological process, was constructed. The functional module of genes identified using gene co-expression network and constructed from microarray expression data with scale-free network topology was proposed [8]. In addition, the PPI data integrated with expression data was proposed to identify the subnetwork biomarker for cancer classification [9–12]. These successful integrations depended on the efficiency of searching and the scoring algorithm [13].

Recently, the Gene-Network-based Feature Set (GNFS), a method that integrates the statistical significant genes from a gene-set-based approach with network topology data, has been proposed [14]. The GNFS method was proposed for gene subnetwork identification to handle both binary class and multiclass expression data by implementing the algorithm of ANOVA-based Feature Set (AFS) method [15]. Originally, the AFS method was proposed for multiclass-based gene-set-based analysis for significant gene identification. The GNFS, however, uses this method to integrate three assumptions for disease gene biomarker identification by focusing on the identification of the significant gene subnetwork for each pathway or gene-set data from gene expression data. Although the GNFS method was successfully used for subnetwork identification, providing better classification performance and biological interpretation, there is still a need to improve the subnetwork biomarker in order to enhance both disease classification performance and biological relevance. The GNFS used a greedy-search approach to obtain the subnetwork by considering only the maximum increased score of the subnetwork. By using this criteria, only the candidate gene having the highest activity score was accounted as a gene member for optimal expansion, thus ignoring candidate genes having a lower score. This causes a loss of informative genes during searching and scoring. The result is a linear topology. Therefore, alternative searching strategies for subnetwork identification to achieve the gene subnetwork was considered.

This work proposed a new method named Gene Sub-Network-based Feature Selection or GSNFS by implementing two alternative searching schemes for improving gene subnetwork identification, namely gene-set-based or GS search and parent-node-based or PN search algorithms. The proposed algorithms would expand the gene subnetwork that aggregates more significant genes within the identified subnetwork. Four lung cancer expression datasets were used as case studies. The classification performance was used for validation of the identified subnetworks compared with the greedy search algorithm used in the GNFS method.

## Methods

### Expression data

Four microarray gene expression datasets of lung cancer used for gene subnetwork identification were downloaded from Gene Expression Omnibus or GEO database (http://www.ncbi.nlm.nih.gov/geo/) [16]. The dataset of GSE18842 (denoted as Lung1) was published by Sanchez-Palencia et al. [17]. There are 91 samples of which 46 are primary adenocarcinoma and squamous-cell carcinoma samples and 45 are non-tumour samples as control samples. The dataset of GSE10072 (denoted as Lung2) was published by Landi et al. [18]. There are 107 samples of which 58 are adenocarcinoma samples and 49 are non-tumour samples as control from tissue samples of adenocarcinoma paired with non-involved lung tissue from current, former and non-smokers. The dataset of GSE4115 (denoted as Lung3) was published by Spira et al. [19]. There are 187 samples of which 90 are smokers without lung cancer and 97 are smokers diagnosed of having cancer. The dataset of GSE7670 (denoted as Lung 4) was published by Su et al. [20]. The 26 samples from lung cancer patient at the Taipei Veterans General Hospital were separated into pairwise samples of 26 adjacent normal part of adenocarcinoma and 26 tumour part of adenocarcinoma in this study.

In this work, each microarray dataset was pre-processed by discarding the microarray probe representing multiple genes in order to avoid the ambiguous interpretation of those genes. The expression values were normalized using z-transformation into standard scores having mean of zero and standard deviation of one for further analysis.

### Gene-set data

The gene-set data were used for gene expression transformation prior to the gene subnetwork identification considering each gene-set or pathway data. The list of genes that correspond to each gene-set or pathway were provided. In this work, the gene-set data from PathwayAPI [7] were used. The PathwayAPI is a web service for retrieving the curated gene-sets data to consider gene-gene relationship. The gene-sets data consist of 319 pathways of 7467 genes.

### Network data

Two sets of network data were used for the purpose of comparison. First, the GGI from PathwayAPI which composed of 7637 genes and 60,853 gene-gene relationships were used as network data in this study. Then to expand on the dataset, a comparison was made with those identified by using protein-protein interaction (PPI) network.

The PPI and genetic interactions data were downloaded from the Biological General Repository for Interaction Datasets (BioGRID) (http://thebiogrid.org/) (version 3.4.136 update April 25th, 2016). The BioGRID, originally proposed by Stark et al. [21], is an open access database that collects genetic and protein interactions curated from the primary biomedical literature proven to exist via small-scale or large-scale experimental methods [22].

The PPI data from BioGRID was pre-processed to obtain only the interactions of human gene and protein (Entrez ID: 9606). The interactions (edges) with self-loop or self-interaction and duplicate edge were removed. After pre-processing, 16,041 genes/proteins with 213,996 interactions, which provides a topology, were used for further study.

### Dataset homogeneity measurement

When using publicly available microarray gene expression data from different experiments, there may be some covariates confound the data space of those datasets. Therefore, to generate a reliable result, it is required to check for their homogeneity. This work applied the measurement of the data homogeneity in order to provide a reliability measure of the datasets used for cross-validation. To determine the relative contribution to sample variation by the disease status and batch effects of merging a microarray dataset from the public database, Engchuan et al. [23] proposed a measure to deal with the limitations in using those microarray datasets by assessing cluster purity regarding disease status (*Purity*_*D*_) and cluster purity regarding batch status (*Purity*_*B*_). The measurement was calculated based on the analysis of the clustering result of the merged dataset. The clustering was done using k-means clustering approach in the R package. For the *k* parameter, it was set as the number of classes of the merged dataset. The purity level shows how cluster members are mixed from different classes or different statuses. Here, the purity was presented in two terms, *Purity*_*B*_ and *Purity*_*D*_, which can be calculated as follows:

$$ Purity\left(\varOmega, C\right)=\frac{\left(\frac{1}{N}{\displaystyle {\sum}_k}ma{x}_j\left|{w}_k{\displaystyle \cap }\ {c}_j\right|\right) \times j-1}{j-1} $$

where Ω = {*w*_*1*_ *, w,*_*2*_ *…, w*_*k*_} is the set of clusters, *C* = {*c*1, *c*2, …, *cj*} is the set of classes or statuses and *N* is the total number of instances. The range of purity value was normalized to be in range 0 to 1. A good analysis result is expected to have low *Purity*_*B*_ and high *Purity*_*D*_ to indicate the compatibility of two datasets of the same disease for further comparison analysis.

### Network-based algorithm

The network-based method named Gene Sub-Network-based Feature Selection (GSNFS) method was proposed and used for subnetwork identification in this study. GSNFS implementing two new searching approaches to modify the previously published GNFS method [14]. It is a method for subnetwork biomarker identification based on the integration of gene expression data, gene-set or pathway data, and network data which can handle both multiclass and binary class of gene expression data. For each gene-set or pathway, Analysis of Variance or ANOVA was applied to identify the significant gene (at 0.05 significant level for this study) for further use in the process of subnetwork identification. The network data was used as a scaffold to infer the interaction of a seed gene to the neighbour in the subnetwork identification step. Top 10% of identified significant genes with the highest number of neighbour genes were selected as seed genes. An F-value of ANOVA, was used in consideration of the growing seed and subnetwork expansion. Each seed gene was grown to its neighbour gene, resulting in the highest F-value. The expansion from a current subnetwork to its neighbour depends on the improvement of the F-value when each candidate gene is temporarily added to the subnetwork compared with the score of the current subnetwork. At each subnetwork expansion, the search of its neighbour and the score of the candidate gene calculation are crucial steps in subnetwork identification. Two termination criteria of subnetwork expansion are whether there is no more available gene neighbours for consideration or no more improvement of the current subnetwork score.

By focusing on the modification, the GSNFS aims to improve the classification performance while still maintaining good gene/gene-set level agreement. The GSNFS includes two new searching strategies (GS and PN) during network expansion to obtain better subnetwork biomarkers. In order to evaluate the proposed strategies, the gene subnetworks identified by using these two proposed strategies and the greedy approach used in GNFS were compared based on the classification performance.

The identified gene subnetworks with at least three gene members were further used for analysis and discussion in the evaluation of the performance of the proposed searching algorithms.

**Fig. 1:**
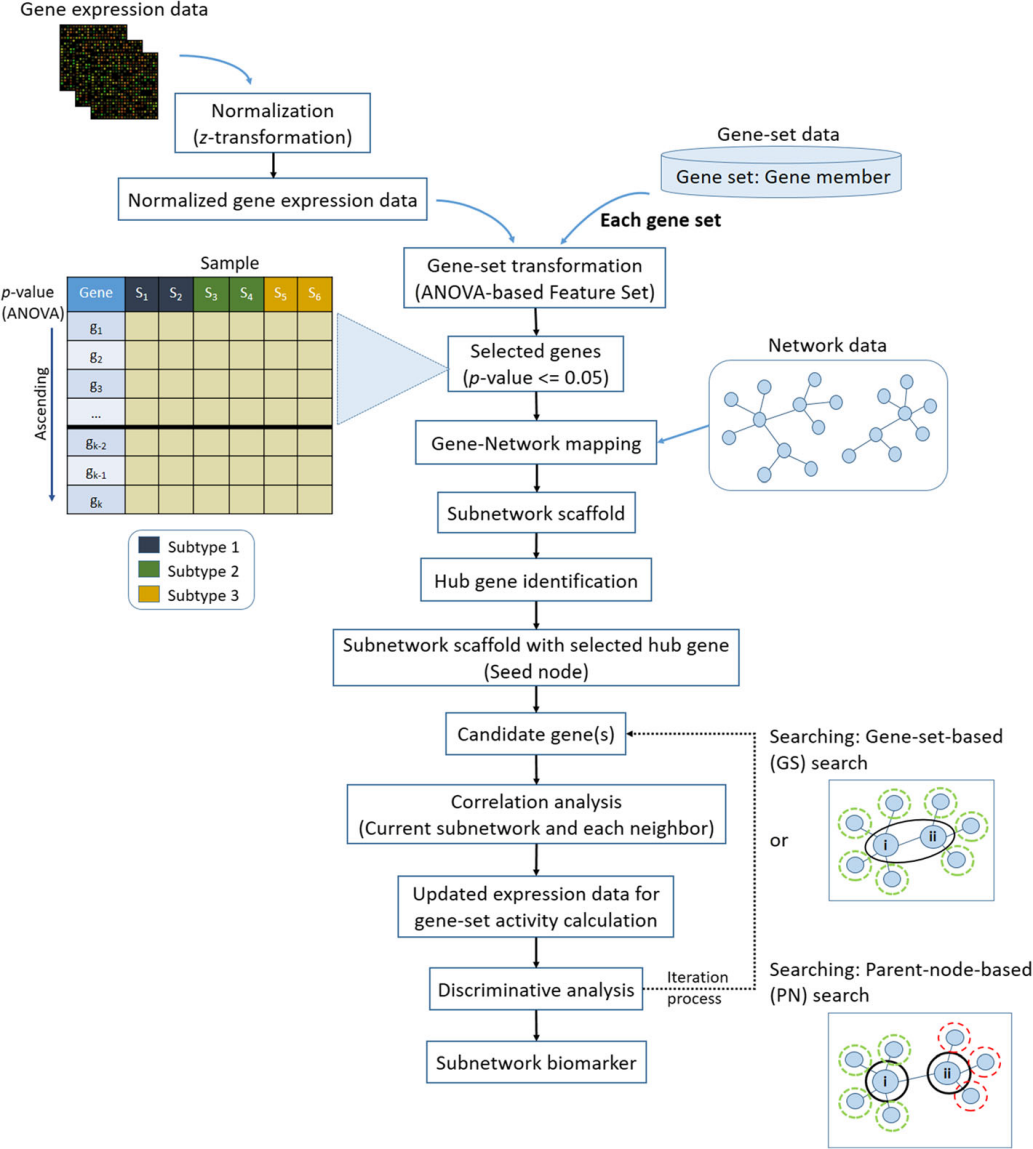
GSNFS framework for gene subnetwork identification. For each gene-set, the expression data was integrated with network data to identify gene subnetwork biomarker for phenotype outcome classification. The improvement of the existing algorithm focused on subnetwork expansion procedure to aggregate significant genes. Two searching methods (GS and PN search) were implemented as searching algorithm in the GSNFS method. GS search treats seed nodes in the i^th^ iteration as a set of the current subnetwork (i, ii) and searches all neighbours of the current subnetwork while PS search only looks for neighbours of a particular gene member in the current subnetwork (i or ii) bypassing genes that are already accounted for the current subnetwork (in this diagram is gene (i) and gene (ii))

The overall steps of the GSNFS method and the modules of searching strategies as proposed in this work are illustrated in Fig. 1.

### Scoring method

The scoring method was used for representing the significance of the subnetwork. It consists of two steps: the correlation analysis and the gene-set activity calculation. The significant genes in each gene-set were obtained using ANOVA for both binary class and multiple class expression data with threshold setting. In correlation analysis, Pearson’s correlation was used to determine the correlation of an activity of a current subnetwork (or seed gene expansion to its neighbour of the first iteration of gene expansion) and a particular candidate expansion gene. The sign (positive or negative) of correlation coefficient was used for determining the criteria to update the activity level of the current subnetwork as explained in [14]. This subnetwork activity represented the summarized expression value of identified subnetwork and was used to calculate the F-value as a subnetwork score. A subnetwork, having the highest F-value of each pathway or gene-set, was selected as the representative of a particular pathway or gene-set.

### Searching algorithm

The GSNFS implements two new searching algorithms: the gene-set-based and parent-node-based approaches as opposed to the GNFS that uses the greedy searching algorithm. The detail of each algorithms are described as follow.

### Gene-set-based (GS) search

This GS search algorithm considered the gene member and the activity score of the current subnetwork. In the i^th^ iteration of subnetwork identification or node expansion, the gene members of a current subnetwork were treated as a set of seed genes to consider the neighbour gene as candidate genes for expansion. All the neighbour genes of this set of seed genes were considered for an expansion of a current subnetwork to the neighbour gene by calculating the F-value. The neighbour gene with maximum F-value was selected and its F-value was compared to the current subnetwork. The expansion was made if there was an improvement in the F-value.

### Parent-node-based (PN) search

The PN search algorithm considered the gene member and the parent gene of a particular candidate gene. As in the i^th^ iteration of subnetwork identification or node expansion, the neighbour genes of all gene members of a current subnetwork were considered as candidate genes for expansion. The F-value between each candidate gene and its interacted gene as the parent node was considered. The current subnetwork expanded to the candidate gene with maximum F-value.

### Greedy search

The greedy search was performed to identify the gene subnetwork in the GNFS method. At each gene expansion level, each neighbour gene of a subnetwork was used to calculate the activity score for comparison with that of the current subnetwork. This comparison was used to consider the improvement when adding that candidate gene to the current subnetwork. By searching for the highest score of each expansion result in local maxima, all neighbours of a current expanded gene were considered to expand to the neighbour gene with the highest score.

### Gene level and gene-set level agreement

The evaluation of the identified gene subnetwork was explored by identifying the co-occurrence of gene or gene-set being identified between two datasets. By doing this, the evaluation was focused on inspecting the presence of the identified gene and gene-set resulting from the analysis without considering the expression value of each gene. The Jaccard-like agreement implemented in Engchuan et al. [24] was used for the evaluation.

The result of the analysis presented the robustness of the proposed method and the agreement of the markers identified from two datasets.

### Cross-dataset validation

The different sets of data generated by different laboratories may contain some confounding factors. As a result, a set of biomarkers identified from one dataset cannot be applied to another. Cross-dataset validation was applied to validate the robustness of a model (gene subnetwork), which is identified by using one dataset as training data and another independent dataset of the lung cancer as test set. This validation process was used to predict the effectiveness of applying the identified subnetwork biomarkers from one dataset (training set) to distinguish the phenotypes of another dataset (test set). The results from cross-dataset validation can reflect the robustness and reliability of an identified biomarker. The classification performance was assessed and presented as Area Under the Curve (AUC) of the Receiver Operating Characteristic curve. The support vector machine (SVM) in WEKA version 3.7-12 with default parameter setting (C-SVC classification with kernel type of radial basis function) was used in this study.

## Results

GSNFS with the GS searching algorithm (GSNFS-GS) and PN searching algorithm (GSNFS-PN) were proposed and implemented in this work. The identified subnetworks obtained from GSNFS using these two new searching strategies were compared with those obtained from the greedy search algorithm originally used in GNFS. Four lung cancer datasets retrieved from GEO database were assessed for their compatibility for further classification evaluation by the purity index analysis (Table 1). A pair of datasets was considered highly compatible if they have low *Purity*_*B*_ and high *Purity*_*D*_. From the result, only two pairs of datasets are compatible. The datasets Lung1 and Lung2 were found to be compatible for comparison analysis with the *Purity*_*B*_ and *Purity*_*D*_ of 0.081 and 0.6, respectively. The datasets Lung2 and Lung4 were compatible with the *Purity*_*B*_ and *Purity*_*D*_ of 0.34 and 0.64, respectively. Lung3 was found to have low comparable manner for classification validation with the other three datasets (Lung1, Lung2 and Lung4) because the assessment results were low in *Purity*_*D*_. In this study, however, the comparison analysis with Lung3 has also been done to assess the ability of the proposed method with the least compatible datasets due to a limited number of datasets resulting from the same assumption of the experiment. The identified gene subnetworks of a particular dataset were obtained using three different searching approaches. The number of gene subnetworks obtained using GGI from PathwayAPI and PPI network is shown in Table 2.

**Table 1:** The homogeneity of datasets

Data	*Purity*_*B*_	*Purity*_*D*_
Lung1*2	0.081	0.6
Lung1*3	0.35	0.12
Lung1*4	0.27	0.34
Lung2*3	0.27	0.14
Lung2*4	0.34	0.64
Lung3*4	0.56	0.029

The homogeneity of each pair of independent datasets was measured and presented in terms of *Purity*_*B*_ and *Purity*_*D*_. The low *Purity*_*B*_ and high *Purity*_*D*_ present the high compatibility of the two datasets for further cross-dataset validation

**Table 2:** The number of identified gene subnetworks using GGI from PathwayAPI and PPI network on different three approaches

Data	GGI			PPI		
	GNFS-greedy [14]	GSNFS-GS	GSNFS-PN	GNFS-greedy [14]	GSNFS-GS	GSNFS-PN
Lung1	65	84	94	40	46	62
Lung2	49	65	92	29	34	42
Lung3	7	4	53	6	11	25
Lung4	16	23	46	14	20	35

The GGI from PathwayAPI and PPI were used as network topology data in the GSNFS method for inferring the interaction among significant genes. Applying the two searching strategies resulted in potential gene subnetworks as disease biomarkers.

Subnetworks having at least three gene members were used to evaluate the agreement of gene/gene-set of the two independent datasets. The gene/gene-set level agreement is a ratio of the number of common identified gene/gene-set from the two datasets and the total number of identified gene/gene-set in the two datasets (Table 3).

**Table 3:** Evaluating the resulting subnetworks based on gene level/gene-set level agreement

Data	GGI			PPI		
	GNFS-greedy [14]	GSNFS-GS	GSNFS-PN	GNFS-greedy [14]	GSNFS-GS	GSNFS-PN
Gene level						
Lung1*2	0.099	**0.138**	0.118	**0.186**	0.140	0.145
Lung1*3	0.023	0.004	**0.068**	0.047	**0.087**	0.085
Lung1*4	0.047	0.084	**0.094**	0.1	0.108	**0.127**
Lung2*3	0.043	0.019	**0.096**	0.081	0.104	**0.107**
Lung2*4	0.091	0.105	**0.121**	0.087	**0.1**	0.076
Lung3*4	0.037	0	**0.084**	**0.143**	0.076	0.104
Gene-set level						
Lung1*2	0.2	0.319	**0.453**	0.23	0.23	**0.316**
Lung1*3	0.058	0.0115	**0.272**	0	0.056	**0.16**
Lung1*4	0.125	0.103	**0.308**	0.08	0.179	**0.366**
Lung2*3	0.037	0.015	**0.321**	0.059	0.125	**0.175**
Lung2*4	0.083	0.219	**0.34**	0	**0.174**	0.167
Lung3*4	0.095	0	**0.2375**	0.111	0.148	**0.2**

The gene/gene-set level agreement is a ratio between the number of common genes/gene-sets found in two datasets and the total number of genes/gene-sets identified from the two datasets. The gene/gene-set level agreements were calculated for different searching strategies and presented above

The cross-dataset validation was used to validate the performance of the identified subnetworks by applying them in disease classification using an independent dataset of the same disease. The classification performance of the identified subnetworks using the GGI from PathwayAPI and PPI network with different searching strategies is shown in Table 4.

**Table 4:** Classification performance of cross-dataset validation using GGI from PathwayAPI and PPI network

Data	GGI			PPI		
	GNFS-greedy [14]	GSNFS-GS	GSNFS-PN	GNFS-greedy [14]	GSNFS-GS	GSNFS-PN
Lung1*2	**0.828**	0.704	0.546	0.634	**0.773**	0.717
Lung1*3	0.54	**0.59**	0.546	**0.568**	0.481	0.535
Lung1*4	0.75	**0.865**	0.635	0.519	0.635	**0.75**
Lung2*1	0.834	**0.868**	0.578	0.724	**0.791**	0.689
Lung2*3	0.479	**0.556**	0.532	0.53	**0.588**	0.537
Lung2*4	**0.865**	0.827	0.692	**0.731**	0.712	0.635
Lung3*1	0.275	0.539	**0.726**	**0.65**	0.607	0.647
Lung3*2	0.378	0.624	**0.729**	0.35	0.526	**0.558**
Lung3*4	0.481	**0.712**	0.635	0.462	**0.673**	0.615
Lung4*1	**0.902**	0.848	0.69	0.563	0.693	**0.857**
Lung4*2	0.805	**0.849**	0.77	0.583	0.72	**0.751**
Lung4*3	0.603	0.549	**0.608**	0.595	0.608	**0.631**

The classification performance of the identified subnetworks resulted from different searching strategies in the subnetwork identification

To compare the effectiveness of gaining biological related genes in the gene subnetworks, the overlapped or common genes among the identified gene subnetworks using different four datasets, three searching strategies, and two networks were inspected as shown in Table 5.

**Table 5:** Common genes found in the identified subnetworks with at least three gene members across four datasets by using three different approaches

GGI			PPI		
GNFS-greedy [14]	GSNFS-GS	GSNFS-PN	GNFS-greedy [14]	GSNFS-GS	GSNFS-PN
MAPK1	None	MAPK1	MAPK1	*EGFR*	*EGFR*
		MAP2K3	TNFRSF1A	MAPK1	MAPK1
		MAP2K4	SHC1	TNFRSF1A	TNFRSF1A
		PRKCA	SOS2	MAP3K7	
			STAT3		

All gene members in gene subnetworks with at least three gene members were inspected for the common genes obtained from the identified gene subnetworks across four datasets by using different searching strategies and different network data in subnetwork identification. The genes in italic indicate the important genes in lung cancer

## Discussion

The homogeneity of between the four datasets was measured to illustrate the difference in distribution of the two independent datasets. Table 1 showed that Lung1, Lung2 and Lung4 datasets are compatible as derived from the low batch effect for *Purity*_*B*_ and high *Purity*_*D*_ in the disease class assessment. Nevertheless, the results measured between Lung1 and Lung3, Lung2 and Lung3, or Lung3 and Lung4 showed low compatibility which can imply the difference between data distribution of Lung3 from the others. Therefore, the classification using the model trained by using either Lung1, Lung2 or Lung4 dataset may be inefficient when applied to classify Lung3 dataset. Thus, in this study, Lung3 was used as cross-dataset validation to validate the performance of the identified subnetworks despite its low compatibility in order to observe the robustness of the identified subnetworks.

The number of identified gene subnetworks obtained from the three approaches; GNFS-greedy search, GSNFS-GS and GSNFS-PN, was different. To obtain significant genes, statistical analysis was applied. The seed genes were identified among those genes for subnetwork expansion. The difference among these three approaches is that it only influences the number of gene members in each gene subnetwork. In Table 2, when considering gene subnetworks with at least three gene members, the number of gene subnetworks varied especially in Lung3 dataset. This may be attributed to the genetic difference in the all-smoker samples, where some have lung cancer while others do not have it. The number of identified subnetworks resulted from Lung1 was different from that of Lung2 and Lung4 because more number of genes being covered by microarray platform used to generate Lung1 dataset. The GSNFS-PN provides the largest number of gene subnetworks in both network data.

The consistency of identified subnetworks of the two independent datasets were evaluated based on the occurrence of the same gene/gene-set in term of gene/gene-set level agreement. From Table 3, the GSNFS-GS and GSNFS-PN have more common genes between datasets. This result can be used for further study of genes or gene-sets consistently presented in the lung cancer biomarkers.

The identified gene subnetworks obtained from these two searching strategies improved the classification performance when compared with the gene subnetworks identified using the greedy search. The classification evaluation performed by applying the subnetworks identified from one dataset to distinguish the sample with disease from the healthy sample on independent datasets. The GSNFS-GS potentially resulted in subnetworks having a better classification performance.

Considering different network data, the PPI network contains more genes/proteins than the GGI network. However, the identified subnetworks obtained by using GGI provided a better gene/gene-set level agreement and classification performance than the subnetworks identified by using PPI. This might be due to the use of subnetworks with at least three gene members for evaluating the gene/gene-set level agreement and the classification performance. The identified subnetworks with one or two gene members were discarded. In addition, the large number of genes/proteins and their interactions in PPI may not imply the function of those genes in term of interacting gene/protein pairs. In contrast, the GGI data provide the relationship of genes having interaction in functional basis based on curated data. This result shows that the relationship of genes in different assumption of network data is important in network-based analysis. After analysing these two network data using Merge Network plug-in of Cytoscape tool (version 3.3.0), there were 3101 nodes found as overlapping gene/protein and 1212 of those having 1929 overlapping interactions. This small number of common genes/proteins and interactions resulted in higher gene level agreement and higher number of overlapping among the four datasets (see Table 5) in the subnetworks using PPI as network data.

From the analysis, the better classification performance was obtained when applying GGI to Lung1, Lung2, and Lung4 datasets. Moreover, when applying the PPI network to the analysis, the subnetworks identified from Lung1 and Lung2 also performed well on the classification analysis.

Table 4 shows that the two proposed searching algorithms increase the performance of the classification when compared with the original greedy search implemented in the GNFS method. However, the GSNFS-PN presented robustness when applied to Lung3 and Lung4 dataset in terms of improving the cross-dataset classification performance and increasing the gene/gene-set level agreement results. Nevertheless, the improvement of the classification performance and gene level agreement was seen by applying the GSNFS-GS to Lung1 and Lung2. This implies that the GSNFS-PN may be suitable with the datasets having low compatibility while the GSNFS-GS could be efficient with datasets having high compatibility. The subnetworks identified from Lung4 dataset using the GSNFS-PN with PPI network performed well when applying to independent datasets. This classification performance of Lung4 was consistent with high number of gene/gene-set level agreement of Lung4 with other three datasets. When comparing between GSNFS-GS and GSNFS-PN in overall, it was found that GSNFS-PN performed better when considering the gene/gene-set level agreement. Meanwhile the GSNFS-GS was better when considering the classification performance analysis.

The effectiveness of the two proposed algorithms for searching in biological meaning is shown in Table 5. The well-studied gene related to lung cancer, *EGFR* gene, [25, 26] was identified by these two searching strategies using PPI network. This gene is shown in italic in Table 5. Moreover, *MAPK1* (mitogen-activated protein kinase 1) gene was found as a common gene among all four lung cancer datasets except for the GSNFS-GS. This gene encodes for a member of the MAP kinase family, which plays a role in a variety of cellular processes such as proliferation, differentiation, transcription regulation and development by controlling phosphorylation at nuclear target. The *TNFRSF1A* (tumour necrosis factor receptor superfamily member 1A) gene was found as a common gene using PPI as the network data. This gene encodes for a protein, which is one of the major receptors for the tumour necrosis factor-alpha. The function of this receptor is related to activate NF-kappaB, mediate apoptosis which may play a role in tumourigenesis. These results illustrated the improvement of identified gene subnetworks using the GSNFS method with the proposed searching algorithms for gene subnetwork biomarker identification.

In this work, each gene expression dataset was pre-processed. The homogeneity measurement of the datasets have been done with the purpose of inspecting the contribution of the data in two independent datasets. Another task of the data pre-processing step was a batch effect correction to reduce the systematic error of generating microarray data from a different batch.

## Conclusions

This work proposed a new network-based method named Gene Sub-Network-based Feature Selection or GSNFS. GSNFS identifies gene subnetwork biomarkers by using two new searching algorithms: gene-set-based search and parent-node-based search algorithms. With the use of our newly proposed searching and scoring algorithms, more numbers of genes were obtained in the subnetwork expansion step. As a result, the evaluation showed the improvement of the identified subnetworks regarding its classification performance and gene/gene-set level agreement over the greedy search algorithm implemented in the GNFS method. Applying the homogeneity measurement to the dataset provided a reliable result for cross-dataset validation. The results from the classification performance and the biological relevance showed that the proposed searching strategies are quite effective in identifying of gene subnetwork as biomarker for lung cancer.
